# Time‐restricted eating and concurrent exercise training reduces fat mass and increases lean mass in overweight and obese adults

**DOI:** 10.14814/phy2.14868

**Published:** 2021-05-27

**Authors:** Christopher J. Kotarsky, Nathaniel R. Johnson, Sean J. Mahoney, Steven L. Mitchell, Regina L. Schimek, Sherri N. Stastny, Kyle J. Hackney

**Affiliations:** ^1^ Department of Health, Nutrition, and Exercise Sciences North Dakota State University Fargo ND USA; ^2^ Department of Radiology Sanford Health Fargo ND USA; ^3^ Department of Health and Human Physiological Sciences Skidmore College Saratoga Springs NY USA

**Keywords:** aerobic training, intermittent fasting, obesity, resistance training, weight loss

## Abstract

The purpose of this study was to determine whether time‐restricted eating (TRE), also known as time‐restricted feeding, was an effective dietary strategy for reducing fat mass and preserving fat‐free mass while evaluating changes in cardiometabolic biomarkers, hormones, muscle performance, energy intake, and macronutrient intake after aerobic and resistance exercise training in physically inactive and overweight or obese adults. This study was a randomized, controlled trial. Overweight and obese adults (mean ± SD; age: 44 ± 7 years; body mass index [BMI]: 29.6 ± 2.6 kg/m^2^; female: 85.7%) were randomly assigned to a TRE or normal eating (NE) dietary strategy group. The TRE participants consumed all calories between 12:00 p.m. and 8:00 p.m., whereas NE participants maintained their dietary habits. Both groups completed 8 weeks of aerobic exercise and supervised resistance training. Body composition, muscle performance, energy intake, macronutrient intake, physical activity, and physiological variables were assessed. A total of 21 participants completed the study (NE: *n* = 10; TRE: *n* = 11). A mild energy restriction was observed for TRE (~300 kcal/day, 14.5%) and NE (~250 kcal/day, 11.4%). Losses of total body mass were significantly greater for TRE (3.3%) relative to NE (0.2%) pre‐ to post‐intervention, of which TRE had significantly greater losses of fat mass (9.0%) compared to NE (3.3%). Lean mass increased during the intervention for both TRE (0.6%) and NE (1.9%), with no group differences. These data support the use of TRE and concurrent exercise training as a short‐term dietary strategy for reducing fat mass and increasing lean mass in overweight and obese adults.

## 1 INTRODUCTION

Developing dietary strategies to maintain muscle mass and strength during weight loss is critical as the prevalence and incidences of obesity and physical dysfunction (e.g., sarcopenia and dynapenia) continue to rise in the US population, along with obesity‐related conditions (e.g., cardiovascular disease, stroke, type 2 diabetes, and certain types of cancers; (Buford et al., 2010; Hales et al., 2017; National Heart, Lung, and Blood Institute, 2014). These conditions represent major health problems and are some of the leading causes of preventable death in the United States (National Heart, Lung, and Blood Institute, 2014). Sarcopenia, the age‐related loss of muscle mass, negatively affects strength, balance, and stability, increasing risk of falls and impairing ability to perform activities of daily living such as walking, personal care, cooking, and chores (Tinetti et al., 2006). Nonetheless, the most alarming consequence of decreased muscle strength is its ability to predict future mortality in middle‐aged and older adults (Cooper et al., 2010).

Fasting, categorized as either long‐term (LTF) or short‐term (STF), is a dietary approach in which individuals abstain from energy intake for periods longer than the typical 8 h of sleep to induce health benefits (Mattson et al., 2017; Paoli et al., 2019; Rothschild et al., 2014). Fasting affects substrate metabolism, numerous cellular pathways, such as lipolysis and autophagy, as well as the cardiovascular system and inflammation (Paoli et al., 2019). LTF, performed 3 days or more, depletes glucose reserves in the body and induces ketosis, whereas STF utilizes fasting durations insufficient to induce ketosis in the body (Paoli et al., 2015, 2019). Intermittent fasting (e.g., alternate‐day fasting or whole‐day fasting 1 to 2 days/week), periodic fasting (i.e., three or more days of fasting every 2 to 3 weeks), and time‐restricted eating (TRE; i.e., ad libitum energy intake within a defined window of time) are all popular STF regimes (Anson et al., 2003; Harvie & Howell, 2017; Paoli et al., 2019; Tinsley & La Bounty, 2015).

The main appeal of STF dietary strategies is that individuals do not need to intentionally restrict energy intake every day, or at all, such as with continuous energy restriction (CER), to attain weight loss and cardiometabolic benefits (Harvie & Howell, 2017). This is important considering CER is associated with poor compliance and appears to accelerate the return of pre‐deprivation body mass levels once the restraints regarding eating are removed (Anastasiou et al., 2015). Also, CER is known for weight loss consisting of 6% to 38% fat‐free mass, which suggests a large proportion of metabolically active skeletal muscle tissue is lost instead of adipose tissue (Chaston et al., 2007). Short‐term fasting, on the other hand, has shown high levels of compliance and does not appear to lead to a compensatory over‐consumption on the non‐dieting days (Gabel et al., 2018; Harvie et al., 2013). In fact, recent studies in young men and women have found that TRE combined with resistance training results in an improvement of muscle performance, a reduction of fat mass, and a maintenance or increase of lean mass (Moro et al., 2016; Tinsley et al., 2017, 2019).

While diet and exercise improve many health consequences of obesity and attenuate declines in muscle mass and strength, dietary strategies are not always followed nor manageable for long‐term use. Fortunately, the highly adhered to STF regimes have shown promise to deliver benefits similar to CER in terms of weight loss and cardiometabolic health (Harris et al., 2018; Harvie et al., 2013; Mattson et al., 2017; Rynders et al., 2019). While TRE has become an increasingly popular STF strategy, there are still a limited number of studies observing its effects on body composition and cardiometabolic health with and without exercise. Concurrent training, the inclusion of resistance and aerobic training in a single exercise program, appears to be the optimal method for improving overall functional ability and physical performance when compared to aerobic or resistance training alone (Villareal et al., 2017; Wilson et al., 2012). Thus, more controlled trials on TRE involving concurrent training are needed to validate the dietary strategy on its ability to preserve fat‐free mass during weight loss and reduce disease risk in adults (Moro et al., 2016; Tinsley et al., 2017, 2019).

The purpose of this study was to determine whether TRE was an effective dietary strategy for reducing fat mass and preserving fat‐free mass while evaluating potential changes in cardiometabolic biomarkers, hormones, muscle performance, energy intake, and macronutrient intake after aerobic and resistance exercise training in physically inactive and overweight or obese adults.

## 2 MATERIALS AND METHODS

### 2.1 Overview

This study was a randomized, controlled trial. The primary outcome measures for this study were fat mass and fat‐free mass. Secondary outcomes included levels of physical activity, muscle performance, blood pressure, blood and saliva markers, and dietary intake. Data collection occurred from October 2018 to December 2019 at North Dakota State University in Fargo, North Dakota, USA. The study was registered at clinicaltrials.gov (NCT03823872) and was approved by the North Dakota State University Institutional Review Board (#HE18247). After providing written informed consent and completing a health history questionnaire and PAR‐Q, documents were screened by the research team to determine whether participants were healthy and capable of participating in the study.

### 2.2 Participants

**FIGURE 1:**
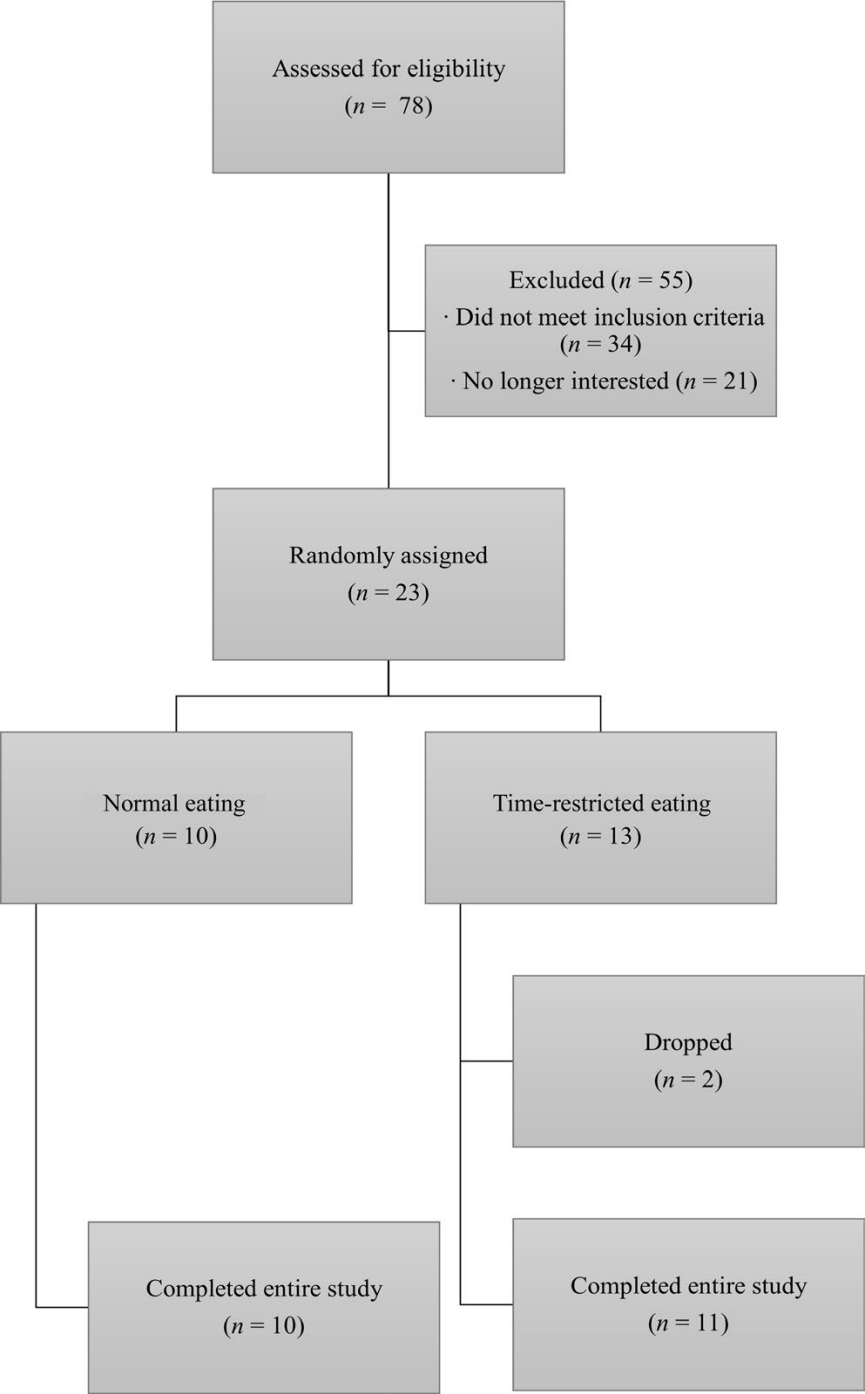
Subject screening and completion flowchart

Physically inactive and overweight or obese female and male participants, determined by a body mass index (BMI) between 25.0 and 34.9 kg/m^2^, between the ages of 35 and 60 years were recruited via email announcements, flyers, and word of mouth. Physically inactive was defined as individuals who are not currently following a structured aerobic or resistance training program (Thivel et al., 2018). Participants were screened and then excluded if they were pregnant, trying to become pregnant, currently smoking tobacco, using e‐cigarettes or smokeless tobacco, had previous injuries that would prevent them from exercising, currently following a structured dietary plan, taking medications that could influence muscle size and strength (e.g., testosterone and growth hormone), had uncontrolled chronic heart related conditions, had any major signs of cardiovascular, pulmonary, or metabolic disease, had two or more major coronary risks factors, or had other reasons needing medical clearance. Of 78 screened participants, 23 were determined eligible to participate in the study and were randomly assigned to a TRE or NE group. During the study, two participants dropped out due to unrelated reasons (i.e., dental complication and back injury not related to the study). A total of 21 participants completed the study (Figure 1).

### 2.3 Dietary strategy

An ad libitum approach was implemented to examine how energy and macronutrient intake are influenced when eating patterns are not strictly controlled. TRE participants were required to consume all their calories between 12:00 p.m. and 8:00 p.m. each day, inducing a fasting window of 16 h, while NE participants were required to maintain their regular eating schedule. An eating window between 12:00 p.m. and 8:00 p.m. was selected to align with previous literature on TRE and resistance training (Moro et al., 2016; Tinsley et al., 2019). All dietary instructions were led by a protocol‐trained, board‐certified specialist in sports dietetics via a previously recorded instructional video. Participants were instructed to eat at self‐selected intervals and to consume their typical diet throughout the day. Participants in the TRE group were immediately transitioned to the 8‐h eating window Week 1, rather than gradually increasing their daily fasting time each week. Participants in TRE were encouraged to consume only water, black coffee, or tea during the 16‐h fasting window. Adverse events related to TRE adaptation were noted by researcher's during Week 4 as part of the safety monitoring session and at post‐intervention assessments Week 8.

### 2.4 Resistance training

Resistance training was standardized for both groups and consisted of three different workouts, performed on non‐consecutive days, each week for 8 weeks. The three resistance training routines were as follows: Workout A (chest press, shoulder press, and triceps pulldown), Workout B (leg press, leg flexion, and leg extension), and Workout C (wide grip latissimus dorsi pulldown, back machine pulley row, and bicep curl). The training protocol involved three sets of 12 repetitions with no more than 60 s of rest between exercises and sets (Baechle et al., 2008). Load was assigned based on a percentage of the participants body mass and was adjusted through trial loads during Week 1 of the intervention, until the goal number of repetitions was achieved (Baechle et al., 2008).

For general muscular fitness, the American College of Sports Medicine (ACSM) recommends untrained individuals resistance train each major muscle group 2 to 3 days/week, with at least 48 h separating sessions for the same muscle group (American College of Sports Medicine, 2017). While training each major muscle group at least twice a week can maximize muscle growth, once a week remains an effective strategy for increasing muscular hypertrophy (Schoenfeld et al., 2016). In fact, it is not required to progressively increase the training stimulus (i.e., adding resistance, sets, or training sessions per week) when the program is designed to maintain current levels of muscular fitness (i.e., strength and mass) as outlined for this study (Aird et al., 2018). Muscular strength may be maintained by training each muscle group as little as 1 day/week if the intensity or the resistance lifted is held constant (American College of Sports Medicine, 2017). This is important, as the current study repetition range (Chaston et al., 2007; Collaborators GBD 2015 Obesity, 2017) falls within the very light‐to‐light intensity of 10 to 15 repetitions (40% to 50% of 1RM) recommended by ACSM for improving strength in very deconditioned or sedentary individuals beginning a resistance training program (American College of Sports Medicine, 2017). This is further supported by a previous TRE study that showed a maintenance of fat‐free mass and maximal strength in healthy, resistance‐trained males following an 8‐week resistance training program in which each major muscle group was trained only 1 day/week (Moro et al., 2016).

For the first exercise of each workout, participants followed a lift specific warm‐up by performing eight repetitions at 40% of their estimated 12RM, followed by a 1‐min rest, and then a second warm‐up set of six repetitions at 60% of their estimated 12RM, followed by a 2‐min rest (Baechle et al., 2008). Participants began Week 2 attempting to complete three sets of 12 repetitions with their adjusted loads. During subsequent training sessions each week, participants attempted to complete one additional repetition for each set. Once participants performed three sets of 15 repetitions, on two consecutive training sessions, intensity was increased by adding weight in 2.27‐ to 9.09‐kg increments. After adding additional weight, participants went back to performing three sets of 12 repetitions. The increase in weight ensured participants maintained appropriate training progression to elicit the desired training response.

Resistance training sessions were performed between 1:00 p.m. and 7:00 p.m. for TRE participants and between 9:00 a.m. and 7:00 p.m. for NE participants to ensure no exercise was completed in a fasted state. Certified personal trainers supervised all routines to ensure proper form and safety. Participants were instructed not to perform any other resistance training exercises outside of their weekly sessions. Due to the complexity of matching participant and trainer schedules, personal trainers were not blinded to participant group allocation.

### 2.5 Aerobic training

ACSM has defined 50 to 60 min/day to total 300 min of moderate, or 150‐min of vigorous, physical activity per week necessary to promote or maintain weight loss (American College of Sports Medicine, 2017). The current study was designed to help and encourage participants to achieve this goal each week. Heart rate maximum was calculated using the Tanaka method (Tanaka et al., 2001), and heart rate reserve (HRR) was used to prescribe aerobic training intensities. Though an incremental exercise test would be a more preferred method for measuring cardiorespiratory fitness and prescribing exercise intensity, the physiologic responses observed would be most relevant to the mode the exercise test was conducted on (e.g., cycle and treadmill) (American College of Sports Medicine, 2017). HRR was selected for prescribing exercise intensity as it is an ACSM recommended method that permits exercise on a variety of aerobic training equipment through heart rate monitoring, rather than metabolic equations that prescribe intensities on specific equipment (American College of Sports Medicine, 2017). This was important as a portion of each participant's weekly physical activity goals were completed on their own time. Thus, HRR was most ideal as it is easily understandable and effective at monitoring exercise intensity anywhere.

The training protocol began at a moderate‐to‐vigorous (≥55% HRR) intensity and was performed on a treadmill or related aerobic conditioning equipment. After completion, participants were encouraged to perform a 5‐min cooldown at a low (<40% HRR) to moderate (40% to 55% HRR) intensity. Participants were instructed to perform a minimum of 75 min of moderate‐to‐vigorous aerobic activity, not including time spent in supervised resistance training, at Week 1, which increased 75 min each subsequent week until Week 5. At Weeks 5 and 7, training intensity was increased (5% or 10%) to maintain exercise progression, depending upon participants conditioning.

Aerobic training was also performed between 1:00 p.m. and 7:00 p.m. for TRE participants and between 9:00 a.m. and 7:00 p.m. for NE participants to ensure no exercise was completed in a fasted state. Aerobic training was completed immediately following resistance training on lifting days and at participant convenient times on non‐lifting days, three or more days per week depending on weekly physical activity goals and aerobic training intensity.

### 2.6 Adherence monitoring

Dietary intake and adherence were measured by 3‐day dietary records (Prentice et al., 2011), which were analyzed using Food Processor software (ESHA) by research assistants and line‐by‐line verified by a registered dietitian. Intake was collected on two typical days and one atypical day at pre‐intervention and Weeks 1, 4, and 7. For TRE, if a participant indicated energy intake outside the eating window for any of the 3 days collected at Weeks 1, 4, and 7, it was flagged as noncompliant. More than 1 day of noncompliance would exclude the participant from the study.

Personal trainers were instructed to record each instance of a participant missing a training session. If a workout was missed, the participant was contacted, and an effort was made to reschedule the training session. More than four missed workouts were considered noncompliant and grounds for the participant's removal from the study. Additional compliance for physical activity was measured through ActiGraph GT3X+ accelerometers (ActiGraph), which were worn at Weeks 2, 5, and 8 to precisely quantify the intensity of participants activities. The accelerometer was worn on the right hip, in line with the right knee, via an elastic band. The accelerometer was worn during waking hours except for water‐based activities. A sleep log was provided to subjects to record the time they removed the accelerometer for sleep and the time they put the device on the following day upon waking.

The GT3X+ accelerometers were processed with epoch durations of 60 s. Participants were required to have worn the accelerometer for at least 4 days, including one weekend day, over a 7‐day collection period, with a minimum of 10 h of wear time each day. The Choi et al. (Choi et al., 2011) algorithm was used for wear and non‐wear time validation. To classify physical activity, Sasaki, John, and Freedson (Sasaki et al., 2011) cut‐points for moderate, vigorous, and very vigorous physical activity were used, along with Kozey‐Keadle, Libertine, Lyden, Staudenmayer, and Freedson (Kozey‐Keadle et al., 2011) cut‐points for assessing sedentary behavior.

### 2.7 Overview of lab assessments

The assessments for this study were conducted by trained research assistants during two separate sessions at pre‐ and post‐intervention. Post‐intervention assessments were scheduled within 1 h of the participants corresponding pre‐intervention assessment. Participants were instructed to wear similar, light, workout attire at pre‐ and post‐intervention assessment sessions. Session 1 was conducted within 1 h of waking, after a 12‐h fast, between 6:00 a.m. and 9:00 a.m. At Session 1, anthropometrics, resting blood pressure and resting heart rate, metabolic and physiological variables, and body composition were assessed. Session 2 was conducted in the afternoon in a nonfasted state to assess muscle performance and submaximal cardiorespiratory fitness. After pre‐training assessments, participants were randomly assigned to the TRE or NE group.

### 2.8 Anthropometric and hemodynamic assessments

Resting blood pressure and heart rate were collected after 5 min of sitting. Blood pressure was measured with a Diagnostix 703 sphygmomanometer (American Diagnostic Corporation, Hauppauge, NY, USA). Heart rate was counted for 1 min at the radial artery by manual palpation. After resting measures, height was measured using a stadiometer (Seca 213), and body mass was recorded (Denver Instrument DA‐150) with shoes removed. A spring‐loaded measurement tape with Gulick attachment (Fabrication Enterprises) was used to measure hip and waist circumference at the widest portion of the hips and at two finger widths above the umbilicus, respectfully.

### 2.9 Metabolic and physiological assessments

Blood and saliva were collected within 1 h of waking between 6:00 a.m. and 9:00 a.m. Capillary blood drops were collected from the fingertip and dried on filter paper as dried blood spots (DBS), as described (Kapur et al., 2008, 2010). Blood spots were dried for at least 4 h, and DBS filter cards were then stored at −80°C until shipment in bulk to ZRT Laboratory for testing. Saliva was collected into plastic tubes by passive drool as described (Dimitrakakis et al., 2010; Glaser et al., 2008). Both DBS and saliva assays were performed by immunoassays as previously described (Dimitrakakis et al., 2010; Glaser et al., 2008; Kapur et al., 2008, 2010). ZRT Laboratory is a CLIA‐approved laboratory. Results of the laboratory analyses were provided to the study investigators.

The blood spot testing was used to analyze potential changes of several health‐related biomarkers (insulin, high‐sensitivity C‐reactive protein, hemoglobin A1c, triglycerides, total cholesterol, high‐density lipoprotein, low‐density lipoprotein, and very low‐density lipoprotein). Dried blood spot testing has shown strong correlation with conventional serum tests, making it a reliable and convenient tool for screening cardiometabolic risk factors (Kapur et al., 2008, 2010).

The saliva was used to analyze potential changes in hormones (estradiol, progesterone, testosterone, dehydroepiandrosterone sulfate, and cortisol). Oral hormone users were told to make sure any night dosage was applied at least 12 h before planned morning collection. Saliva testing has been shown to be reliable for hormone analysis (Dimitrakakis et al., 2010; Glaser et al., 2008; Gröschl, 2008).

### 2.10 Body composition assessment

Measurements of body composition were taken via dual‐energy X‐ray absorptiometry (DXA) on a Lunar Prodigy, Model #8915 (GE Healthcare), with enCORE software. Quality assurance was established each morning prior to a scan using a quality control block. Participants were positioned according to manufacturer's recommendations. Female participants were screened for pregnancy with a urinary human chorionic gonadotropin test (ClinicalGuard) to confirm that each participant was not pregnant before scanning. Researchers noted the position of any participant who did not fit within the scanning dimensions to ensure the same limbs were estimated during the mirroring scan procedure at both pre‐ and post‐intervention assessments. DXA was conducted during Session 1, after an overnight fast, in rested participants, as scans are effective at identifying total and lean mass changes in response to the quantity and amount of a given meal consumed (Nana et al., 2015).

### 2.11 Muscle and aerobic performance assessments

All subjects completed a low‐to‐moderate warm‐up on a Monark 828E cycle ergometer (Monark Exercise AB), for 5 min before testing. Handgrip strength was measured in kilograms using a Jamar Hydraulic Hand Dynamometer (Sammons Preston Rolyan). Participants were asked to stand with their dominant arm bent at 90°. After a 3‐s countdown, participants squeezed the dynamometer as hard as possible, with encouragement, for an additional 3 s, followed by 1 min of rest. The test was repeated two more times, and the greatest of three attempts was recorded.

To observe potential changes in cardiorespiratory fitness due to aerobic conditioning throughout the study, a 3‐min step test was conducted according to the protocol designed by the YMCA (American College of Sports Medicine, 2017). Because the main research aims were fat mass loss and fat‐free mass preservation, not cardiorespiratory improvement, the YMCA test was chosen as it personifies a functional testing capability and is simplistic in quantifying cardiorespiratory performance in terms that are understandable for participants.

A Biodex Pro4 system dynamometer (Biodex Medical Systems) was used to measure lower body muscle strength and endurance of the right leg. Isokinetic maximal strength was defined as peak torque and assessed at 60° per second for the knee extension–flexion and 30° per second for plantar–dorsiflexion. Isokinetic endurance was defined as total work and measured using a 21 repetitions test at 180° per second for the knee extension–flexion and 60° per second for plantar–dorsiflexion.

### 2.12 Safety monitoring

At Week 4, participants' resting blood pressure and body mass were measured to verify safe weight loss. Safe weight loss was defined as 1 to 2 lb/week, or about 2 to 8 lb/month. As it is common to experience greater weight loss during the start of a new weight loss program, researchers were responsible for determining whether weight loss was too drastic compared to safe weight loss values. Additionally, researchers noted any adverse events experienced by the participants while adapting to the TRE dietary strategy. At post‐intervention assessments Week 8, previously reported events were followed up with and new adverse events were recorded.

### 2.13 Statistical analysis

An a priori power analysis was performed with G*Power software Version 3.1.9.3. (Heinrich Heine University Düsseldorf) using an effect size (ES) estimated from a previous investigation of TRE and resistance training (Moro et al., 2016). Fat mass was specified as the primary dependent variable for the power analysis, and the ES used for the power analysis was Cohen's *f* (Cohen's *d* divided by 2). Cohen's *d* was calculated by dividing the difference between the mean change in fat mass for the TRE group and the mean change in fat mass for the control group by the pooled standard deviation. Using this ES (*f* = 0.405), an *α* error probability of 0.05, and a power of 0.8, it was estimated that 16 participants were needed to detect significant changes in fat mass. Therefore, to promote adequate power and account for a 25% attrition rate, the minimum target sample size was 20.

For body composition, cardiometabolic biomarker, hormone, muscle performance, and cardiovascular performance variables, separate 2 (dietary plan: TRE and NE) by 2 (time: pre‐ and post‐intervention) ANOVAs with repeated measures were used. For physical activity variables, separate 2 (dietary plan: TRE and NE) by 3 (time: Weeks 2, 5, and 8) ANOVAs with repeated measures were used. For dietary intake variables, separate 2 (dietary plan: TRE and NE) by 4 (time: pre‐intervention and Weeks 1, 4, and 7) ANOVAs with repeated measures were used. An *α* level of 0.05 was used to determine statistical significance. If a significant interaction was found, independent and paired *t* tests with Bonferroni corrections were run to compare post‐training adaptations. If independent *t* tests failed to show the significant interaction effect, delta differences for the variables were calculated, and independent *t* tests were used to remove between participant variability. Cohen's *d* ES was calculated for each group by dividing the difference between pre‐ and post‐intervention by the pooled standard deviation. All statistical analyses were conducted using SPSS 26 software (IBM Corp.).

## 3 RESULTS

### 3.1 Participants

A total of 21 participants (NE: *n* = 10; TRE: *n* = 11) completed the study. The NE group was composed of nine female participants and one male participant, while the TRE group contained nine female participants and two male participants. No baseline differences were present between the two groups (Table 1).

**TABLE 1:** Participant characteristics at baseline

	NE (*n* = 10)	TRE (*n* = 11)	*p* (group)
Age (years)	44 ± 2	45 ± 3	0.739
Body mass (kg)	83 ± 3	82 ± 3	0.823
Height (cm)	168 ± 3	166 ± 2	0.599
BMI (kg/m^2^)	29.4 ± 0.8	29.8 ± 0.8	0.742
Hip circumference (cm)	108.4 ± 2.9	109.2 ± 1.6	0.806
Waist circumference (cm)	95.3 ± 3.0	98.0 ± 1.9	0.445

Note

Data are presented as mean ± standard error; *p* values are from one‐way analysis of variance.

Abbreviations: BMI, body mass index; NE, normal eating; TRE, time‐restricted eating.

A time by group effect was observed for BMI and body mass from pre‐ to post‐intervention. Post hoc tests for BMI and body mass showed significant decreases for TRE. Group delta differences in BMI and body mass from independent *t* tests showed significant decreases in TRE relative to NE. A significant time effect was observed for waist circumference pre‐ to post‐intervention (Table 2).

**TABLE 2:** Anthropometrics

Group	Pre‐intervention	Post‐intervention	Δ	ES (*d*)	*p* (group)	*p* (time)	*p* (I)
Body mass (kg)							
NE	83 ± 3	83 ± 3	0	−0.04	0.641	**0.003**	**0.025**
TRE	82 ± 3	79 ± 3	−3	−0.30			
BMI (kg/m^2^)							
NE	29.4 ± 0.8	29.3 ± 0.9	−0.1	−0.04	0.966	**0.005**	**0.019**
TRE	29.8 ± 0.8	28.8 ± 0.8	−1.0	−0.36			
Hip circumference (cm)							
NE	108.4 ± 2.9	108.9 ± 2.7	0.5	0.07	0.886	0.328	0.079
TRE	109.2 ± 1.6	107.2 ± 1.7	−2.0	−0.35			
Waist circumference (cm)							
NE	95.3 ± 3.0	92.3 ± 3.0	−3.0	−0.32	0.626	**0.018**	0.493
TRE	98.0 ± 1.9	92.8 ± 2.2	−5.2	−0.77			

Note

Data are presented as mean ± standard error; *p* values are from repeated‐measures analysis of variance. The bold emphasis was used to indicate (bring attention to) statistical significance within the table.

Abbreviations: BMI, body mass index; ES, effect size; I, interaction; NE, normal eating; TRE, time‐restricted eating.

### 3.2 Physical activity

**FIGURE 2:**
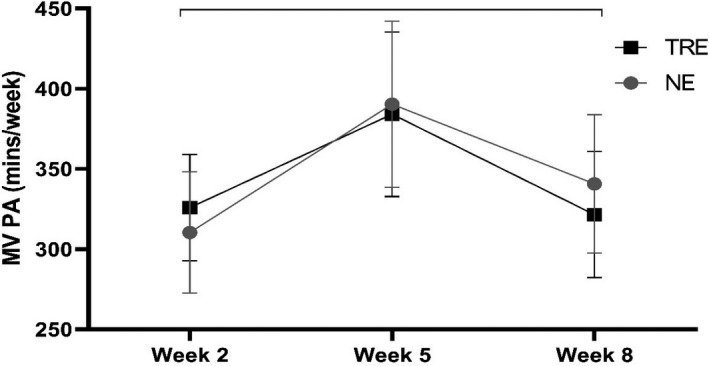
Average time spent in moderate‐to‐vigorous (MV) physical activity (PA). Results display (time‐restricted eating [TRE]: *n* = 11; normal eating [NE]: *n* = 10) average minutes (mean ± SE) at Weeks 2, 5, and 8. Brackets indicate a significant change within groups (i.e., time main effects), with no significant differences between groups

There was a significant time effect observed for step count and moderate‐to‐vigorous physical activity across the intervention (see Table 3, which shows physical activity data; https://figshare.com/s/1a6f87ce7c6105b47422). Average time spent in moderate‐to‐vigorous physical activity across the intervention was above 300 min for both groups (Figure 2).

**TABLE 3:** Physical activity

Group	Week 2	Week 5	Week 8	*p* (group)	*p* (time)	*p* (I)
Sedentary time (min/day)						
NE	2985 ± 184	2992 ± 192	2767 ± 295	0.648	0.464	0.252
TRE	2905 ± 233	2653 ± 164	2839 ± 113			
Light PA (min/day)						
NE	2429 ± 198	2559 ± 200	2296 ± 264	0.455	0.439	0.251
TRE	2315 ± 101	2200 ± 157	2246 ± 116			
MV PA (min/day)						
NE	310 ± 38	390 ± 51	341 ± 43	0.952	**0.019**	0.782
TRE	326 ± 33	384 ± 51	326 ± 39			
Steps (#/day)						
NE	7541 ± 782	8781 ± 842	8039 ± 686	0.686	**<0.001**	0.299
TRE	8116 ± 828	9773 ± 1186	7929 ± 835			

Note

Data are presented as mean ± *SE*. *p* values are from repeated‐measures ANOVA. Bold emphasis was used to indicated (bring attention to) statistical significance (i.e., an alpha level of 0.05) within the tables.

Abbreviations: I, Interaction; MV, moderate‐to‐vigorous; NE, normal eating; PA, physical activity; TRE, time‐restricted eating.

### 3.3 Dietary intake

A significant time effect was observed for energy intake across pre‐intervention and Weeks 1, 4, and 7. Significant time effects were also observed for carbohydrate intake in both g/day and g/kg/day across the same period (Table 4).

**TABLE 4:** Dietary intake

Group	Pre‐intervention	Week 1	Week 4	Week 7	Δ	*p* (group)	*p* (time)	*p* (I)
Energy (kcal)								
NE	2227 ± 177	1985 ± 158	1921 ± 158	1974 ± 245	−253	0.467	**0.003**	0.510
TRE	2112 ± 179	1640 ± 159	1856 ± 208	1806 ± 180	−306			
Protein								
(g)								
NE	76 ± 8	71 ± 6	76 ± 9	70 ± 10	−6	0.899	0.088	0.611
TRE	83 ± 9	68 ± 6	73 ± 8	73 ± 7	−10			
(%)								
NE	14 ± 1	14 ± 1	16 ± 1	14 ± 1	0	0.106	0.208	0.347
TRE	16 ± 1	17 ± 1	16 ± 1	16 ± 1	0			
(g/kg)								
NE	0.9 ± 0.1	0.8 ± 0.1	0.9 ± 0.1	0.8 ± 0.1	−0.1	0.624	0.111	0.532
TRE	1.0 ± 0.1	0.8 ± 0.1	0.9 ± 0.1	0.9 ± 0.1	−0.1			
Carbohydrate								
(g)								
NE	266 ± 18	241 ± 19	236 ± 19	235 ± 26	−31	0.209	**0.001**	0.240
TRE	255 ± 29	177 ± 24	202 ± 22	190 ± 28	−65			
(%)								
NE	49 ± 4	50 ± 3	50 ± 3	49 ± 3	0	0.107	0.336	0.316
TRE	48 ± 3	43 ± 3	44 ± 3	41 ± 2	−7			
(g/kg)								
NE	3.2 ± 0.2	2.9 ± 0.2	2.9 ± 0.2	2.8 ± 0.2	−0.4	0.230	**0.001**	0.215
TRE	3.1 ± 0.3	2.2 ± 0.3	2.5 ± 0.3	2.4 ± 0.3	−0.7			
Fat								
(g)								
NE	83 ± 10	76 ± 9	71 ± 8	77 ± 11	−6	0.961	0.407	0.602
TRE	82 ± 9	68 ± 7	79 ± 13	81 ± 8	−1			
(%)								
NE	33 ± 3	34 ± 2	33 ± 2	34 ± 2	1	0.056	0.299	0.785
TRE	35 ± 2	37 ± 2	37 ± 2	41 ± 1	6			
(g/kg)								
NE	1.0 ± 0.1	0.9 ± 0.1	0.8 ± 0.1	0.9 ± 0.1	−0.1	0.719	0.316	0.493
TRE	1.0 ± 0.1	0.8 ± 0.1	1.0 ± 0.2	1.0 ± 0.1	0.0			

Note

Data are presented as mean ± standard error; *p* values are from repeated‐measures analysis of variance. The bold emphasis was used to indicate (bring attention to) statistical significance within the table.

Abbreviations: I, interaction; NE, normal eating; TRE, time‐restricted eating.

### 3.4 Body composition

**FIGURE 3:**
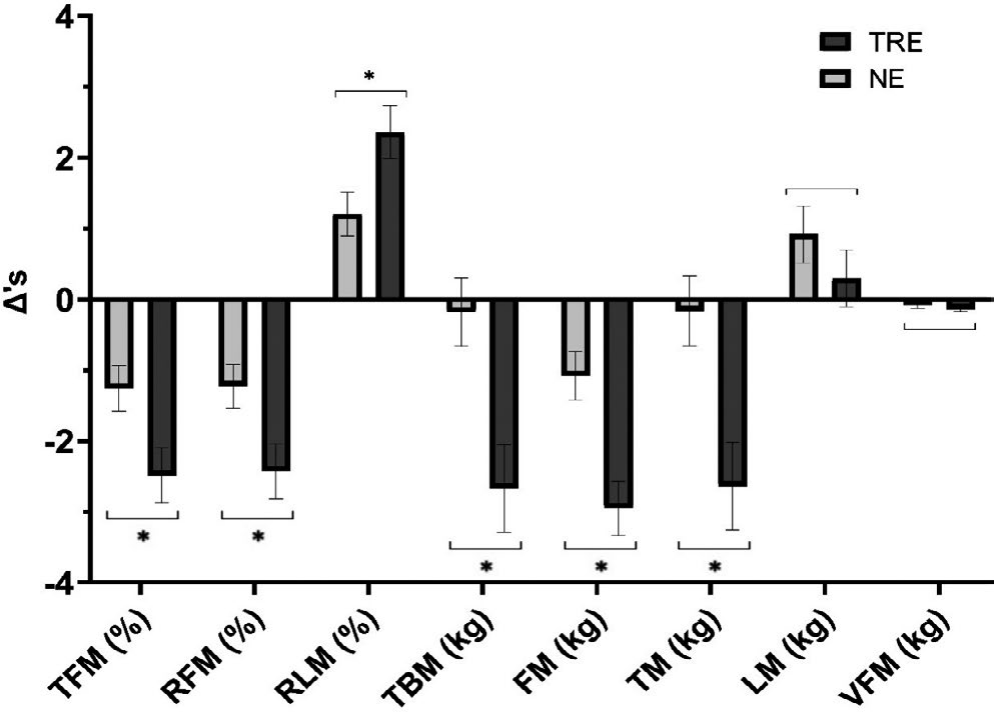
Body composition changes. Results display (time‐restricted eating [TRE]: *n* = 11; normal eating [NE]: *n* = 10) delta differences (mean ± SE) between pre‐ and post‐intervention. Asterisks with brackets indicate a significant difference in TRE relative to NE (i.e., interaction effect). Brackets indicate a significant change within groups (i.e., time main effects), with no significant differences between groups. FM, fat mass; LM, lean mass; RFM, region fat mass; RLM, region lean mass; TBM, total body mass; TFM, tissue fat mass; TM, tissue mass; VFM, visceral fat mass

A time by group interaction effect was observed for tissue and region fat mass (%), region lean mass (%), fat mass (kg), tissue mass (kg), and total body mass (kg) from pre‐ to post‐intervention. Post hoc tests for tissue and region fat mass, region lean mass, and fat mass showed significant decreases for NE and TRE. Post hoc tests for tissue mass and total body mass showed significant decreases for TRE. Group delta differences in tissue and region fat mass, fat mass, tissue mass, and total body mass from independent *t* tests showed significant decreases in TRE relative to NE, while region lean mass showed a significant increase in TRE relative to NE. A significant time effect was observed for lean mass and visceral fat mass pre‐ to post‐intervention (Figure 3). Interestingly, TRE visceral fat mass decreased almost twice as much relative to NE (see Table 5, which shows body composition data; https://figshare.com/s/d7a2857f5456489cf94c).

**TABLE 5:** Body composition

Group	Pre	Post	Δ	ES (d)	*p* (group)	*p* (time)	*p* (I)
Tissue fat mass (%)							
NE	41 ± 2	40 ± 2	−1	−0.20	0.902	**<0.001**	**0.026**
TRE	41 ± 2	39 ± 2	−2	−0.43			
Region fat mass (%)							
NE	40 ± 2	38 ± 2	−2	−0.20	0.887	**<0.001**	**0.026**
TRE	40 ± 2	37 ± 2	−3	−0.43			
Region lean mass (%)							
NE	57 ± 2	59 ± 2	2	0.20	0.917	**<0.001**	**0.029**
TRE	57 ± 2	59 ± 2	2	0.44			
Tissue mass (kg)							
NE	80 ± 3	80 ± 3	0	−0.02	0.612	**0.002**	**0.006**
TRE	79 ± 2	77 ± 3	−2	−0.32			
Fat mass (kg)							
NE	33 ± 2	32 ± 2	−1	−0.14	0.746	**<0.001**	**0.002**
TRE	33 ± 2	30 ± 2	−3	−0.44			
Lean mass (kg)							
NE	47 ± 2	48 ± 2	1	0.12	0.707	**0.046**	0.292
TRE	47 ± 2	47 ± 2	0	0.06			
Total body mass (kg)							
NE	83 ± 3	82 ± 3	−1	−0.02	0.624	**0.002**	**0.006**
TRE	82 ± 3	79 ± 3	−3	−0.32			
Visceral fat mass (kg)							
NE	1.07 ± 0.17	1.00 ± 0.15	−0.07	−0.14	0.700	**0.001**	0.261
TRE	1.01 ± 0.18	0.87 ± 0.16	−0.14	−0.25			
BMC (kg)							
NE	2.58 ± 0.13	2.57 ± 0.12	−0.01	−0.03	0.915	0.119	0.221
TRE	2.60 ± 0.11	2.58 ± 0.11	−0.02	−0.05			
BMD (g/cm^2^)							
NE	1.23 ± 0.04	1.24 ± 0.04	0.01	0.09	0.751	0.387	0.161
TRE	1.26 ± 0.04	1.25 ± 0.04	−0.01	−0.10			

Note

Data are presented as mean ± *SE*. *p* values are from repeated‐measures ANOVA. Bold emphasis was used to indicated (bring attention to) statistical significance (i.e., an alpha level of 0.05) within the tables.

Abbreviations: BMC, bone mineral content; BMD, bone mineral density; ES, effect size; I, Interaction; NE, normal eating; TRE, time‐restricted eating.

### 3.5 Muscle performance

A significant time by group interaction effect was observed for knee extension strength peak torque pre‐ to post‐intervention. Group delta differences in knee extension strength peak torque from an independent *t* test showed a significant increase in NE relative to TRE. However, post hoc tests for knee extension strength peak torque revealed no significant changes for the TRE or NE group. Significant time effects were observed for knee flexion strength peak torque and endurance total work pre‐ to post‐intervention. Significant time effects were also observed for dorsiflexion strength peak torque and endurance total work pre‐ to post‐intervention (Table 6).

**TABLE 6:** Muscle performance

Group	Pre‐intervention	Post‐intervention	Δ	ES (*d*)	*p* (group)	*p* (time)	*p* (I)
Handgrip (kg)							
NE	43 ± 4	44 ± 4	1	0.12	0.601	0.573	0.341
TRE	41 ± 3	41 ± 3	0	−0.04			
KE STR PT (Nm)							
NE	138 ± 7	156 ± 7	18	0.83	0.523	0.220	**0.014**
TRE	140 ± 15	134 ± 13	−6	−0.14			
KF STR PT (Nm)							
NE	82 ± 6	100 ± 5	18	1.01	0.677	**<0.001**	0.636
TRE	80 ± 8	95 ± 7	15	0.61			
KE END TW (J)							
NE	1456 ± 121	1467 ± 152	11	0.02	0.265	0.383	0.301
TRE	1314 ± 144	1190 ± 126	−124	−0.28			
KF END TW (J)							
NE	1109 ± 128	1307 ± 98	198	0.55	0.993	**0.005**	0.773
TRE	1087 ± 120	1325 ± 79	238	0.71			
PF STR PT (Nm)							
NE	86 ± 6	83 ± 5	−3	−0.16	0.109	0.952	0.379
TRE	68 ± 6	71 ± 9	3	0.13			
DF STR PT (Nm)							
NE	20 ± 3	25 ± 2	5	0.72	0.740	**0.043**	0.870
TRE	21 ± 3	26 ± 2	5	0.58			
PF END TW (J)							
NE	760 ± 80	698 ± 82	−62	−0.24	0.231	0.082	0.802
TRE	628 ± 84	547 ± 94	−81	−0.27			
DF END TW (J)							
NE	110 ± 34	231 ± 30	121	1.19	0.730	**0.002**	0.159
TRE	161 ± 45	212 ± 30	51	0.40			

Note

Data are presented as mean ± standard error; *p* values are from repeated‐measures analysis of variance. The bold emphasis was used to indicate (bring attention to) statistical significance within the table.

Abbreviations: DF, dorsiflexion; END, endurance; ES, effect size; I, interaction; KE, knee extension; KF, knee flexion; NE, normal eating; PF, plantar flexion; PT, peak torque; STR, strength; TRE, time‐restricted eating; TW, total work.

### 3.6 Cardiorespiratory performance and hemodynamics

Significant time effects were observed for resting heart rate and step heart rate (i.e., heart rate 1 min after step test completion) pre‐ to post‐intervention. Interestingly, the decrease in TRE step heart rate (−22 bpm) was almost twice as much as NE (−13 bpm) pre‐ to post‐intervention (see Table 7, which shows cardiorespiratory performance and hemodynamics data; https://figshare.com/s/05150079dae5619913a9).

**TABLE 7:** Cardiorespiratory performance and hemodynamics

Group	Pre	Post	Δ	ES (d)	*p* (group)	*p* (time)	*p* (I)
Systolic BP							
NE	120 ± 2	118 ± 3	−2	−0.20	0.921	0.150	0.388
TRE	122 ± 3	116 ± 3	−6	−0.69			
Diastolic BP							
NE	83 ± 1	79 ± 2	−4	−0.87	0.820	0.107	0.168
TRE	81 ± 2	80 ± 2	−1	−0.07			
Resting HR							
NE	75 ± 2	69 ± 3	−6	−0.72	0.654	**<0.001**	0.064
TRE	75 ± 2	66 ± 2	−9	−1.21			
Step HR							
NE	125 ± 6	112 ± 7	−13	−0.63	0.346	**<0.001**	0.205
TRE	122 ± 5	100 ± 6	−22	−1.32			

Note

Data are presented as mean ± *SE*. *p* values are from repeated‐measures ANOVA. Bold emphasis was used to indicated (bring attention to) statistical significance (i.e., an alpha level of 0.05) within the tables.

Abbreviations: BP, blood pressure; HR, heart rate; ES, effect size; I, Interaction; NE, normal eating; TRE, time‐restricted eating.

### 3.7 Metabolic and physiological variables

No significant effects were observed in blood pre‐ to post‐intervention (Table 8). It is important to note that triglycerides, low‐density lipoprotein, and very low‐density lipoprotein were excluded from analysis due to an unknown contaminate on the blood spot cards from the manufacturer that interfered with the enzymes used to detect triglycerides, causing the triglyceride values to be two to three times higher than normal. Both low‐density lipoprotein and very low‐density lipoprotein were impacted as triglyceride values were required for these calculations. Due to hormonal differences between males and females, saliva markers were analyzed using only female participants between NE (*n* = 9) and TRE (*n* = 9). No significant effects were observed in these saliva markers pre‐ to post‐intervention (Table 9).

**TABLE 8:** Resting cardiometabolic profiles

Group	Pre‐intervention	Post‐intervention	Δ	ES (*d*)	*p* (group)	*p* (time)	*p* (I)
Insulin (µIU/ml)							
NE	13 ± 2	10 ± 1	−3	−0.51	0.361	0.092	0.934
TRE	11 ± 1	9 ± 2	−2	−0.44			
hsCRP (mg/L)							
NE	1.0 ± 0.3	1.3 ± 0.3	0.3	0.29	0.114	0.694	0.197
TRE	2.9 ± 0.9	2.4 ± 0.8	−0.5	−0.17			
HbA1c (%)							
NE	4.7 ± 0.2	4.3 ± 0.2	−0.4	−0.70	0.816	0.055	0.142
TRE	4.6 ± 0.2	4.6 ± 0.3	0.0	−0.07			
Cholesterol (mg/dl)							
NE	200 ± 12	199 ± 12	−1	−0.01	0.800	0.804	0.655
TRE	202 ± 12	206 ± 10	4	0.11			
HDL (mg/dl)							
NE	55 ± 6	55 ± 4	0	0.00	0.966	0.589	0.859
TRE	55 ± 3	54 ± 3	−1	−0.43			

Note

Data are presented as mean ± standard error; *p* values are from repeated‐measures analysis of variance.

Abbreviations: ES, effect size; HbA1c, hemoglobin A1c; HDL, high‐density lipoprotein cholesterol; hsCRP, high‐sensitivity C‐reactive protein; I, interaction; NE, normal eating; TRE, time‐restricted eating.

**TABLE 9:** Resting hormonal profiles (female participants only)

Group	Pre‐intervention	Post‐intervention	Δ	ES (*d*)	*p* (group)	*p* (time)	*p* (I)
Estradiol (pg/ml)							
NE	0.8 ± 0.2	1.0 ± 0.2	0.1	0.20	0.467	0.228	0.700
TRE	0.7 ± 0.1	0.9 ± 0.1	0.2	0.72			
Progesterone (pg/ml)							
NE	26 ± 12	17 ± 6	−9	−0.32	0.625	0.181	0.676
TRE	26 ± 12	27 ± 13	1	0.02			
Testosterone (pg/ml)							
NE	23 ± 3	20 ± 3	−3	−0.33	0.433	0.090	0.791
TRE	27 ± 7	25 ± 7	−2	−0.15			
DHEAS (ng/ml)							
NE	4.6 ± 1.2	4.2 ± 0.7	−0.4	−0.13	0.631	0.589	0.165
TRE	4.7 ± 1.0	5.6 ± 1.4	0.9	0.24			
Cortisol (ng/ml)							
NE	5.9 ± 0.8	5.1 ± 0.5	−0.8	−0.38	0.950	0.664	0.418
TRE	5.4 ± 1.0	5.7 ± 0.6	0.2	0.10			

Note

Data are presented as mean ± standard error; *p* values are from repeated‐measures analysis of variance.

Abbreviations: DHEAS, dehydroepiandrosterone sulfate; ES, effect size; I, interaction; NE, normal eating; TRE, time‐restricted eating.

### 3.8 Adherence

The average eating window for TRE was between 12:00 p.m. and 7:30 p.m. (mean ± SD; 7.0 ± 0.8 h) and between 8:00 a.m. and 8:30 p.m. (mean ± SD; 11.9 ± 1.4 h) for NE. Only two instances of dietary noncompliance were reported among two participants in the TRE group throughout the study, indicating a high level of compliance. Overall, no major adverse events were reported following the TRE dietary strategy during the study. At Week 4, ~91% of TRE participants reported no adverse effects. Reported events included morning headaches (*n* = 1). At Week 8, ~91% of TRE participants reported no side effects. The side effects for Week 8 were the same as Week 4 and for the same participant. Adherence to the resistance training program was 100%, with all participants completing their 24 required resistance training workouts for the study. Average time spent in moderate‐to‐vigorous physical activity at Weeks 2, 5, and 8 was above 300 min for both groups, indicating an achievement of weekly physical activity goals.

## 4 DISCUSSION

The purpose of our investigation was to examine the effects of ad libitum TRE and concurrent exercise training on body composition, cardiometabolic biomarkers, hormones, muscle performance, energy intake, and macronutrient intake. The primary findings of this study were that 8 weeks of TRE (i.e., consuming all energy intake between 12:00 p.m. and 8:00 p.m. daily) and concurrent exercise training reduced total body mass, BMI, and fat mass more significantly than NE and the same concurrent exercise training in physically inactive and overweight or obese adults. Secondary findings were that concurrent exercise training significantly increased lean mass and knee flexion and ankle dorsiflexion muscle strength and endurance for TRE and NE, while demonstrating a significant improvement in knee extensor strength peak torque for NE relative to TRE.

Our findings indicated a mild, yet significant energy restriction for the TRE, ~300 kcal/day (14.5%), and NE, ~250 kcal/day (11.4%), groups pre‐intervention to Week 7. Despite these similar reductions in energy intake, our study demonstrated a greater loss of total body mass for TRE (3.3%) relative to NE (0.2%). These reductions in total body mass are similar to previous studies on TRE in middle‐aged and older adults (Anton et al., 2018, 2019; Gabel et al., 2018), though often indicated to be a result of subsequent reductions in energy intake (Gabel et al., 2018; Gill & Panda, 2015). Considering our results display this energy restriction in both groups, it is more probable that the unintentional reduction was induced by the concurrent exercise training or because of increased self‐awareness due to recording of dietary intake. Importantly, losses of fat mass for TRE (9.0%) were nearly three times greater than the NE group (3.3%); suggesting that the metabolic effects of TRE may have a greater impact on the loss of fat mass than mild energy restriction alone, considering that energy and macronutrient intake were not significantly different between the two groups.

Fasting between 12 and 36 h a day has been shown to stimulate fat metabolism and the metabolic switch between glucose oxidation and fat oxidation as glycogen stores are depleted (Collaborators GBD 2015 Obesity, 2017; Paoli et al., 2019). This is accompanied by an increase of triacylglycerol and adipose tissue lipolysis, highlighted by the increase of plasma free fatty acids and glycerol (Paoli et al., 2019; Soeters et al., 2012). Increased lipolysis creates a shift toward fatty acid mobilization and utilization within adipocytes, increasing energy expenditure, which can help protect against obesity (Ahmadian et al., 2009; Wang et al., 2008). Fasting, similar to a ketogenic diet, also induces the phosphorylation of AMP‐activated protein kinase (AMPK) pathway, which affects gene expression and is involved with regulating mitochondrial biogenesis, substrate utilization, including glucose and fatty acid uptake and oxidation, and autophagy (Aird et al., 2018; Mihaylova & Shaw, 2011; Paoli et al., 2019). Autophagy “mediates protein degradation, organelle turnover, and recycling of cytoplasmic components” and is essential for reducing cellular stress and preserving normal cell function, as well as maintaining normal cardiovascular function in the heart and blood vessels (Paoli et al., 2019).

Similarly, these metabolic adaptions that occur during fasting dietary strategies are activated in response to fasted exercise. For example, fasted exercise has shown to mobilize and promote free fatty acid utilization and activate skeletal muscle signaling pathways, including AMPK, when compared to fed exercise (Aird et al., 2018; Guerra et al., 2010). Other posited mechanisms include upregulation in the mRNA expression of key lipolytic enzymes (i.e., adipose triglyceride lipase and hormone‐sensitive lipase), which could help mobilize lipids from adipose tissue (Aird et al., 2018; Chen et al., 2017; Lampidonis et al., 2011). These mechanisms all help promote lipid utilization during fasted exercise (Aird et al., 2018). In fed conditions, mechanistic target of rapamycin pathway (mTOR) is stimulated and helps promote anabolic processing during increased energy availability (Paoli et al., 2019). As such, the philosophy of TRE is to maximize lipid utilization through prolonged fasting hours, while inducing anabolic processing and lean mass preservation through exercise during fed conditions. However, significant gaps exist regarding the effects of fasted compared with fed exercise on skeletal muscle and adipose tissue metabolism, particularly following long‐term interventions. This is especially true for observing these effects during unconventional dietary conditions, such as LTF and STF, during fasted or fed exercise.

The preservation of lean mass while following STF has produced mixed results, as regimes tend to incorporate or induce deficits in energy intake (Catenacci et al., 2016; Tinsley & La Bounty, 2015). Recent studies, however, have demonstrated the ability of TRE and resistance training to at least preserve lean mass and muscle performance (Moro et al., 2016; Tinsley et al., 2017, 2019). We found that lean mass increased across time and, when considering fat mass loss, the percentage of regional lean mass increased to a greater extent in TRE (2.4%) relative to NE (1.2%). The significant interaction effect observed for knee extension strength peak torque showed an increase in NE (13.5%) relative to TRE (−4.8%), suggesting that TRE may negatively affect performance. However, post hoc tests revealed no significant difference in knee extensor peak torque between groups. Moreover, no significant differences between groups were found for any other lower body muscle strength or endurance measure. Thus, the lower knee extensor strength observed in the TRE group does not provide good evidence that TRE worsens lower body strength. Our study supports the use of resistance exercise as a method for increasing lean mass and at least maintaining muscle performance, even while under a mild energy restriction. These findings are particularly important due to the loss of muscle mass associated with aging and in response to energy restriction, which can lead to the loss of functional independence later in life (Buford et al., 2010; Chaston et al., 2007; Tinetti et al., 2006).

Average time spent in moderate‐to‐vigorous physical activity across the intervention was above 300 min for both groups. While this indicates an achievement of weekly physical activity goals, the addition of aerobic exercise did not appear to further enhance weight loss or improve indicators of cardiometabolic health, other than heart rate, relative to studies on aerobic exercise alone (Kraus et al., 2002; Lin et al., 2015). Intermittent fasting and TRE have shown various positive effects on the cardiovascular system, including enhanced parasympathetic activity (mediated by the neurotransmitter acetylcholine) in the autonomic neurons that innervate the heart and arteries, resulting in a reduced heart rate and blood pressure (Longo & Mattson, 2014; Mager et al., 2006; Paoli et al., 2019). With that said, many of the cardiometabolic effects observed in STF remained relatively unaffected by TRE in this study (Harris et al., 2018; Mattson et al., 2017; Rothschild et al., 2014; Rynders et al., 2019). Many of the participants in the present study were metabolically healthy at baseline with all variables at or within the normal range. Research has shown physiological markers in metabolically healthy, obese adults remain relatively unaffected by STF and other dietary regimes (Gabel et al., 2018; Rynders et al., 2019), but further investigation is needed to verify whether individuals with more adverse metabolic profiles receive greater benefits when following STF regimes (Rynders et al., 2019).

The present study also explored the effects of TRE and concurrent exercise training on anabolic and catabolic hormone concentrations. Recent investigations on TRE and resistance training have indicated no change in biological markers in young women and counterintuitive reductions in anabolic hormones (i.e., testosterone and IGF‐1) in resistance‐trained men after 8‐week interventions (Moro et al., 2016; Tinsley et al., 2019). After a 12‐week resistance training intervention in overweight and obese adults, Roberts, Croymans, Aziz, Butch, and Lee (Roberts et al., 2013) saw no decrease in total testosterone, but a decrease in basal testosterone attributed to increased concentrations of sex hormone‐binding globulin. Roberts et al. (Roberts et al., 2013) also found decreases in basal cortisol, which they attributed to an improvement in metabolic profile, specifically basal insulin, even though the obese participants were metabolically healthy before the intervention. It is still unclear whether obese individuals need to perform resistance training at similar intensities as lean individuals to stimulate these anabolic and catabolic hormones (O'Leary & Hackney, 2014). The current study appears to suggest that the unchanged hormonal profiles observed were due to the primarily metabolically healthy female population.

This study has several limitations. First, dietary intake and adherence to TRE were assessed by dietary intake logs. While our study indicated a high level of adherence, it is possible that participants feigned compliance at assessments. Similarly, estimates of nutrient intake may be inaccurate as the faults of using self‐reported dietary intake logs are well recognized (Kirkpatrick et al., 2019). A second limitation is the inability to have complete control over the dietary intake of each group, as the resources required are extensive for closed‐eating studies. Third, while salivary hormone analysis is reliable, a single saliva sample may not have been sufficient for an accurate measure of these hormonal values pre‐ and post‐intervention. Cortisol alone exhibits a very clear awakening response, which leads to substantially different values over the course of the hour after waking (Stalder et al., 2016). Fourth, due to availability of resources and the time frame of the study, personal trainers and research assistants were not blinded to participant group allocation. Lastly, the duration of our study was only 8 weeks. Longer studies are required to determine the degree of weight loss and cardiometabolic improvement that can be achieved with TRE and concurrent exercise training. Future research should explore TRE during fasted versus fed exercise, immediately before eating windows and during fasting windows, with and without amino acid supplementation throughout prolonged, post‐exercise fasting windows.

In summary, these findings suggest an 8‐h TRE window with concurrent exercise training greatly reduces fat mass relative to a NE control group and increases lean mass in physically inactive and overweight or obese adults. While no changes in physiological variables were seen in this study, TRE and concurrent exercise training appear to improve resting heart rate and heart rate recovery after exercise greater than NE. These data support the use of TRE and concurrent exercise training as a short‐term dietary strategy for reducing fat mass and increasing lean mass in physically inactive and overweight or obese adults.

## CONFLICT OF INTERESTS

No conflicts of interest, financial, or otherwise are declared by the authors.

## AUTHOR CONTRIBUTIONS

*Research design*: Christopher J. Kotarsky, Steven L. Mitchell, Sherri N. Stastny, and Kyle J. Hackney. *Data collection*: Christopher J. Kotarsky, Nathaniel R. Johnson, and Sean J. Mahoney. *Data processing*: Christopher J. Kotarsky, Regina L. Schimek, and Sherri N. Stastny. *Statistical analysis*: Christopher J. Kotarsky and Nathaniel R. Johnson. *Drafted manuscript*: Christopher J. Kotarsky. *Edited manuscript*: Christopher J. Kotarsky, Nathaniel R. Johnson, Sean J. Mahoney, Sherri N. Stastny, and Kyle J. Hackney. *Revised manuscript*: Christopher J. Kotarsky. *Approved final manuscript*: Christopher J. Kotarsky, Nathaniel R. Johnson, Sean J. Mahoney, Steven L. Mitchell, Regina L. Schimek, Sherri N. Stastny, and Kyle J. Hackney.
